# Enzymatic hydrolyzation of *Cordyceps militaris* mushroom extracts and its effect on spent hen chicken

**DOI:** 10.5713/ab.23.0518

**Published:** 2024-04-26

**Authors:** Farouq Heidar Barido, Puruhita, Bayu Setya Hertanto, Muhammad Cahyadi, Lilik Retna Kartikasari, Joko Sujiwo, Juntae Kim, Hack-Youn Kim, Aera Jang, Sung Ki Lee

**Affiliations:** 1 Faculty of Animal Science, Universitas Sebelas Maret, Surakarta, Jawa Tengah 57126, Indonesia; 2 Halal Research Center and Services (HRCS), Institute for Research and Community Service, Universitas Sebelas Maret, Surakarta, Jawa Tengah 57126, Indonesia; 3 Restu Dwi Pangan Co, Tangerang, Banten 15345, Indonesia; 4 Department of Animal Production, Faculty of Animal Science, Universitas Gadjah Mada, Sleman, Special Region of Yogyakarta 55281, Indonesia; 5 Department of Biosystems Machinery Engineering, College of Agricultural and Life Science, Chungnam National University, Daejeon 34134, Korea; 6 Department of Animal Resources Science, Kongju National University, Yesan 32439, Korea; 7 Department of Applied Animal Science, Kangwon National University, Chuncheon 24341, Korea

**Keywords:** *Cordyceps militaris*, Hydrolyzation, Protease, Spent Hen Chicken, Texture Profile

## Abstract

**Objective:**

This study was aimed to investigate the effect of fresh and dried hydrolyzed *Cordyceps militaris* (CM) mushroom with proteolytic enzymes; bromelain (CMB), flavorzyme (CMF), and mixture of bromelain: flavorzyme (CMBF) on quality properties of spent hen chicken.

**Methods:**

Mushroom extract (CME) were combined with three proteolytic enzyme mixtures that had different peptidase activities; stem bromelain (CMB), flavorzyme (CMF), and mixture of stem bromelain:flavorzyme (CMBF) at (1:1). The effect of these hydrolysates was investigated on spent hen breast meat via dipping marination.

**Results:**

Hydrolyzation positively alters functional properties of CM protease. in which bromelain hydrolyzed group (CMB) displayed the highest proteolytic activity at 4.57 unit/mL. The antioxidant activity had a significant increment from 5.32% in CME to 61.79% in CMB. A significantly higher emulsion stability index and emulsification activity index compared to CME were another result from hydrolyzation (p<0.05). Texture properties along with the shear force value and myofibrillar fragmentation index were notably improved under CMB and CMBF in fresh condition. Marination with CM mushroom protease that was previously hydrolyzed with enzymes was proven to also increase the nucleotide compounds, indicated by higher adenosine 5′-monophosphate (AMP) and inosine 5′-monophosphate (IMP) in hydrolysate groups (p<0.05). The concentration of both total and insoluble collagen remained unchanged, meaning less effect from CM protease.

**Conclusion:**

This study suggested the hydrolyzation of CM protease with bromelain or a mixture of bromelain:flavourzyme to significantly improve functional properties of protease and escalate the taste-related nucleotide compounds and texture profiles from spent hen breast meat.

## INTRODUCTION

The stiffness of meat is produced from a complex cross-linking reaction between actin and myosin; two myofibrillar protein that together form a tough muscle characteristic known as actomyosin [1]. Another contributing factor to tough texture is aging, which also increases the production of heat-stable collagen [2]. Studies suggested that the actomyosin dissociation [1] and the breakdown of key proteins, like troponin-T, nebulin, dystrophin, titin, and desmin, lead to considerable improvement in postmortem tenderness [3]. Their alterations are orchestrated by the intricate interplay of endogenous proteolytic enzymes (calpains, cathepsin-B, and caspase-3 enzymes) during postmortem periods [4], by initiating the protein hydrolysis reactions.

The attempts to positively modify the meat tenderness can be divided into three basic procedures that uses chemical, physical, and enzymatic approaches. The enzymatic tenderization of meat by plant proteases is one of the widely suggested methods that is gaining popularity due to its minimal side effects and low cost [5]. Actin and myosin are two examples of proteins with meat-toughening capabilities that are degraded by cysteine, serine, and metalloproteases levels within plant extracts [6]. Apart from being approved as generally recognized as safe (GRAS) by the United States Food and Drug Administration (USFDA), plant proteases are also often preferred over proteases derived from microorganisms. This is because plant protease is thought to be safe with little chance of toxicity, easily accessible in the surroundings, and functional throughout a wide range of temperature, pH, and substrates [7].

Mushroom is widely utilized commodities to provide significant improvement for flavor and taste in wide variety of food products. Its unique characteristics for having abundant content of macronutrients including polysaccharides and proteins, as well as micronutrients such as calcium, magnesium, phosphates, vitamins, and bioactive compounds are the reason for its broad functionalities for quality and health improvement [8,9]. Our previous study demonstrated the mushroom from family of *Cordycipitaceae*, particularly that of *Cordyceps militaris* (CM) mushroom exhibited superior properties for enhancing physicochemical attributes and texture profiles of meat-based products [8,10,11]. CM mushrooms are reported to contain diverse metabolites including adenine, adenosine, cordycepine, cordyheptapeptide, and polysaccharides that function as antifatigue, anti-inflammatory, immunomodulatory, and even anti-cancer. Concerning the texture enhancing properties, the adenosine 5′-monophosphate (AMP) [12] and moderate serine- and metalloprotease enzyme activities [13] accounts for its notable tenderization effects.

It is necessary to look into the idea of using potential serine-and metalloprotease from CM mushroom as an alternative to meat tenderizer. Additionally, it has potential as flavor enhancer from the abundance of free amino acids, bioactive compounds, adenosine, and adenosine 5′-monophosphate (AMP). On the other hand, with the the possible modification of kinetic parameters from the enzyme-to-enzyme interaction; a situation that permits the structural alteration of protein sequences. Protease hydrolysis with endo- or exo-peptidases was reported to intervene in its functional qualities through a more diversified amino acid development [14]. In recent years, study on the utilization of enzymes for hydrolization gaining significant interests. It serves to break down long-chain protein sequences and organisms’ cell wall to produce an improved qualities, functionalities, and processing efficiency products [12]. Specifically in meat products, enzymatic hydrolysation are commonly used to promote an improved tenderness and flavour profiles [4,10,11]. With the above mentioned premises, this study aimed to investigate various extracts prepared from CM mushroom by reacting with other proteases (bromelain and flavorzyme) via enzymolysis on texture improvement of spent hen breast meat.

## MATERIALS AND METHODS

### Sample preparation

The CM mushroom extracts were produced according to the methods by Sukkhown et al [15] and Barido and Lee [8] with minor modification. The selection of raw mushroom condition (fresh vs dried) was based on the single factorial design at specific time and temperature. The preparation of CM mushroom was initiated by mixing finely ground mushroom with deionized water at (1:2, w/v) using food blender (EBR9814W; Electrolux, Stockholm, Sweden) at 13,500×g for 1 min. The mixtures were then placed at a controlled temperature of 4°C±2°C. The process took 24 h to obtain a crude CM mushroom extract (CME). Following filtration using Whatman filter paper no. 1, filtered CME were thought to contain the protease isolated from CM mushroom.

With possibility from enzyme-to-enzyme reaction, the mushroom slurries were combined with three proteolytic enzyme mixtures that had different peptidase activities; stem bromelain (CMB), flavorzyme (CMF), and mixture of stem bromelain:flavorzyme (CMBF) at (1:1), and then incubated in a water bath (BW-20G; Biotechnical Services, North Little Rock, AR, USA) at 55°C for 20 h [14]. Prior to the addition of proteolytic enzymes, the pH adjustment was set at 6.5 and the addition amount at 1% of the dry matter base [16]. The extract solutions were centrifuged (1248R; Labogene, Lynge, Denmark) at 1,370×g, 4°C for 30 min, filtered using whatman paper no. 1, gradually placed into freezer til reach −70°C temperature for 24 h, and brought into freeze dryer (FDU-1200; Eyela, Tokyo, Japan) to dry into powder. Each CM protease were prepared in triplicate.

In order to understand the tenderization effect of enzymolyzed CM mushrooms, dipping marination were employed against spent hen breast samples that were formed into 2.5× 2.5×2 cm^3^. A total of eighty six 75 weeks old spent hen chicken were obtained from local market, while the concentration of marination solution was determined based on previously conducted single factorial experiment (Data not shown). Following dipping marination (1 hour, 2°C±2°C), the marinated samples were brought into low-density polyethylene (LDPE) and determined for its structural alteration and nucleotides.

### Proximate composition

Proximate composition of extracts was determined following procedure by the Association of Official Analytical Chemists [17]. The total moisture percentage of the sample was determined by keeping extracts inside the oven at 105°C for 24 h. The Soxhlet ether extraction system was used to calculate crude fat percentage. Meanwhile, the nitrogen content was determined using the Kjeltec system (2200 Kjeltec Auto Distillation Unit; Foss, Hilleroed, Sweden), wherein the percentage was estimated by multiplying total nitrogen with a constant 6.25. The total crude ash content was obtained after burning samples within the muffle furnace at 550°C for 12 h.

### Instrumental color

The visual evaluation of the CM mushroom protease powder was performed using colorimeter measurement (Chroma meter CR-400; Konica Minolta Sensing Inc., Osaka, Japan) with five replications for each sample. A white plate was used as the reference for the chromameter calibration (2° observer; Illuminant C: Y = 93.6, x = 0.3134, y = 0.3194).

### Yield percentage and degree of hydrolysis

The yield percentage of CME was estimated according to the ratio between mass before and after enzymolysis. The yield was determined as the percentage of dry matter content using the formula of Y = (m1/m2)×100, where m1 is the weight in grams of the CM mushroom protease and m2 is the weight in grams of the mushroom that was used. The industry uses the degree of hydrolysis (DH) as a key measure for establishing the functional quality and properties of CME to regulate and track hydrolytic response.

Since higher DH indicates a higher percentage of intact protein being broken down, high score for DH is highly favourable. This study ascertained the CME DH according to formol titration procedure. In brief, 5 g of the CME powder were added with HPLC grade water, adjusted to neutral pH 7.0 using 1 M NaOH according to procedure by Sukkhown et al [15], and an alliquot of 10 mL of 38% formaldehyde (CH_2_O) solution (v/v) was introduced and let to stand for 5 min. mixtures were added with 1% of phenolphthalein functioning as color indicator in titration using 0.1 M of the NaOH. The amount of 0.1 M NaOH that was used for titration was measured and entered into formula to produce DH.

### pH value and total soluble solid

A pH meter (SevenEasy pH; Mettler-Toledo GmbH, Schwerzenbach, Switzerland) that had previously been calibrated was used to test the pH of stirred slurry samples that had been prepared by combining 3 g of sample with 27 mL of deionized water in a homogenizer (PH91; SMT Co., Ltd., Tokyo, Japan). The total soluble solid was measured by using refractometer (PAL-27S; Atago Co., Ltd., Tokyo, Japan) in triplicate.

### Proteolytic activity

Proteolytic activity of CME was calculated by using casein as the substrate [17]. An increase of 0.1 absorbance units at 280 nm after 60 min of incubation at 35°C (MIR-153; Sanyo Electric Co., Ltd., Osaka, Japan) was considered to be one unit of caseinolytic activity.

### 2,2-Diphenyl-1-picrylhydrazy radical scavenging activity

According to a technique by Islam et al [18], 2,2-diphenyl-1-picrylhydrazyl (DPPH) was determined with a minor modification. Briefly, a reaction mixture containing 1 mL of 0.15 mM DPPH-methanol solution, 4 mL of methanol, and 2 μL of test samples (10/mL), butylated hydroxyanisole (BHA), butylated hydroxytoluene (BHT), or deionized water (control) was incubated at room temperature for 30 min and the absorbance was measured at 517 nm using a spectrophotometer (UV-mini 1240 PC; Shimadzu Corp., Kyoto, Japan). The measurement of DPPH was repeated three times for each samples. Obtained results were expressed as percentage (%) of the DPPH radical scavenging activity.

### Emulsifying capacity

The determination of both emulsifying activity index (EAI) and emulsion stability index (ESI) was performed according to the method by Giménez [19] with a minor modification. One mililiter of soybean oil and 3 mL of the extract solution at different concentrations were mixed and homogenised at a speed of 36,000×g for 1 min (T25 Basic; Ultra-Turrax, Werke GmBH & Co., Staufen, Baden-Wurttemberg, Germany). An aliquot of 100 mL emulsion were taken from the bottom of container at 0 and 10 min after homogenization, followed by dilution into 10 mL of 0.3% sodium dodecyl sulfate (SDS) solution (1:200 dilution). The absorbances were measured at 500 nm. The absorbances measured immediately (A0) and 10 min (A10) after emulsion formation.

### Shear force value and myofibrillar fragmentation index

The marinated samples were packed into plastic bags and subjected to boiling in a water bath (Jeio Tech Co., Ltd., Daejeon, Korea) at 75°C for 35 minutes. The shear force values of the samples were measured using a TA-XT2i Plus instrument (Stable Micro Systems, Surrey, UK) with pre-test speed: 2.0 mm/s; test speed: 1.0 mm/s; post-test speed: 10 mm/s. Each sample was evaluated in three replications.

The determination of the myofibrillar fragmentation index (MFI) was performed according to the method described by Barido et al [20] with slight modifications. Each of the marinated samples was prepared in triplicate. To ensure the elimination of visible fat and connective tissue, the sample was minced into a smaller size, and fat was removed. After hydrolysis with a precooled isolating buffer, the absorbance of the sample supernatant was measured at 540 nm by using a UV spectrophotometer (UV-mini 1240 PC; Shimadzu Corp., Kyoto, Japan). The detected optical density was multiplied by 200, and the result was defined as the MFI.

### Adenosine 5′-monophosphate and inosine 5′-monophosphate

The method for analyzing the 5′-nucleotide contents (adenosine monophosphate, inosine monophosphate) was according to the method described by Jayasena et al [21] with slight modifications. The determination of 5′-nucleotides was conducted via an HPLC (Waters, Milford, MA, USA) set with a 4.6×150 mm C18 HPLC column (Agilent Technologies, Santa Clara, CA, USA) equipped with a diode array detector (DAD) at a wavelength of 254 nm. The 5′-nucleotide concentrations are expressed as mg of compound per 100 g of cooked matter (mg/100 g).

### Total and insoluble collagen contents

The total and insoluble collagen contents were determined according to the method described by Jayasena et al [21]. Sample hydrolysis was determined based on the method described by Palka and Daun [22]. The determination of the hydroxyproline content in the sample hydrolysate was calculated via comparison with the standard curve; thus, the result of multiplying the hydroxyproline content by 7.25 was determined as the collagen content and is expressed as mg/g. The extraction of insoluble collagen was performed according to the method described with minor modifications.

### Statistical analysis

Two-way analysis of variance was used to analyze the obtained data using R version 3.6.1 (The R-foundation for Statistical Computing, Vienna, Austria). Statistical significance was set at p≤0.05 and Duncan’s multiple range tests were used to constantly examine each group’s significances.

## RESULTS

### Proximate composition

The basic nutritional content of the CM mushroom were investigated by this study, and the findings are shown in Table 1. The CM mushroom utilized by this study contained 77.43% moisture, 1.01% crude fat, 28.55% crude protein, and 5.30% ash. This falls within the parameters of what [23] previously reported in CM mushroom’s fruiting body. After being treated to enzymolysis and freeze drying, the moisture percentage of the CM mushroom extract powder was drastically reduced and ranged from 28.15% to 29.71%. The crude protein was notably increased at around 30.11% to 30.97%, crude fat content was 0.96% to 1.42%, and the ash percentage was at 4.99% to 5.33%. Regardless of the enzymes used in the enzymatic hydrolysis, it led to increased protein content of CM mushroom.

### Instrumental color

The color changes were also assessed and shown in Table 2. Regardless of the utilized enzyme, the treated samples’ lightness value (L*) and redness value (a*) both significantly decreased and increased during the enzymatic hydrolysis process (p<0.05). However, the utilized enzymes had a less impact on the yellow value of the sample.

### Yield percentage and degree of hydrolysis

The percentage of CM mushroom protease produced after enzymatic hydrolysis process were determined according to the procedure described by Akram et al [24]. As shown in Table 3, the type of raw mushroom used had a notable impact on the yield percentage (p<0.05). When compared to the dried one, the fresh CM mushroom produced a substantially larger percentage of protease powder. Additionally, both in fresh and dried condition, CMB had the highest yield percentage among treatments, followed by CMBF, CMF, and CME (p<0.05). The DH has a significant impact on the protease powder yield following freeze-drying [15]. Similar to the DH (Table 3), this study discovered a synergistic effect of both raw material formand utilized enzymes.

### pH value and total soluble solid

This study discovered a significant falls in pH value as a result from the enzymatic hydrolysis process. As can be seen in Table 3, all of the enzymolysis-treated groups had significantly lower pH values when compared to that of the crude one (p<0.05). With respect to the utilized enzyme, CM mushroom protease that was hydrolyzed with bromelain showed the significantly lower value (5.31) compared to flavorzyme (5.42) and the combination of both bromelain and flavorzyme (5.37). However, there were no appreciable variations (p>0.05) in the raw CM mushroom material used in fresh and dried conditions. Contrary, the enzymolysis process produced a greater total soluble solid (4.83 to 8.73°Brix) compared to that of the crude extract (3.83 to 4.13°Brix). In addition, the CM mushroom extract that was hydrolyzed with bromelain yielded in protease powder with notably higher °Brix compared to that of flavorzyme and mixed of bromelain and crude extract (p<0.05).

### Proteolytic activity

Table 4 displays the enzymatic activities of the CM mushroom extracts. A solution containing CMB protease powder accounted for 4.57 and 2.38 unit/mL of enzyme activity in fresh and dried sample respectively, indicated a notable proteolytic activity in the fresh one. However, the outcome was considerably different to papain, which had the robust maximum activity at 9.61 unit/mL (p<0.05). The enzyme activity was also significantly influenced by the different enzymes utilized to hydrolyze CM mushroom extract, with papain from papaya latex (Sigma Aldrich, Burlington, MA, USA), CMB, CMBF, CMF, and CME having the highest and lowest level of activity, respectively (p<0.05). The highest enzymatic activity was identified for papain with 9.61 unit/mL, wherein the lowest was recorded for the CME with 2.90 unit/mL under fresh condition.

### 2,2-Diphenyl-1-picrylhydrazyl radical scavenging activity

**Figure 1:**
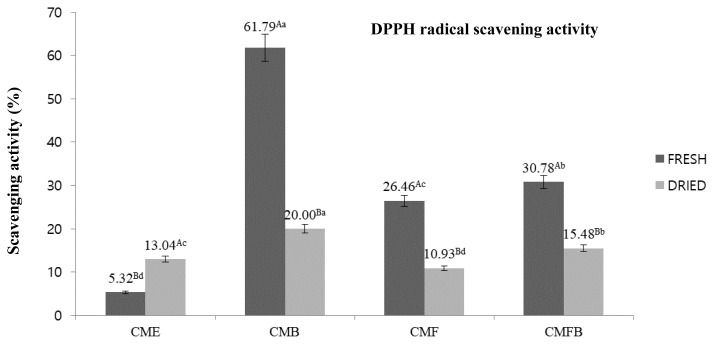
Antioxidant activity of the *Cordyceps militaris* mushroom extract hydrolyzed with various enzymes. CME, *Cordyceps militaris* mushroom extract; CMB, *Cordyceps militaris* mushroom extract hydrolyzed with bromelain; CMF, *Cordyceps militaris* mushroom extract hydrolyzed with flavorzyme; CMBF, *Cordyceps militaris* mushroom extract hydrolyzed with a mixture of bromelain:flavorzyme. ^a–d^ Mean values with different superscripts are significantly different among *Cordyceps militaris* mushroom extract treatments (p<0.05). ^A,B^ Mean values with different superscripts are significantly different between *Cordyceps militaris* mushroom condition (p<0.05).

As seen in Figure 1, the CMB combined with fresh raw materials were characterized having the highest antioxidant activity among samples, showing 61.79% scavenging activity toward DPPH. In addition, the fresh mushroom condition resulted in CM extract powder with the exceptionally greater antioxidant activity compared to that of the dried one, regardless of the utilized enzymes, apart from CME. The antioxidant activity in the CME was ranging from 5.32% to 13.04%, in which CME prepared from the dried CM mushroom showed a notably better scavenging activities.

### Emulsifying capacity

**Figure 2:**
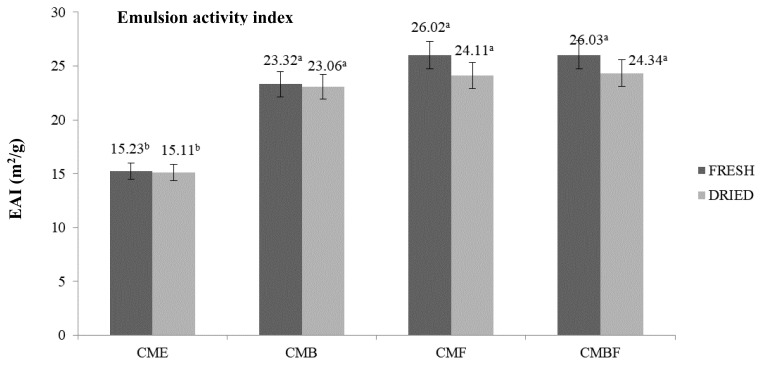
Emulsion activity index of the *Cordyceps militaris* mushroom extract hydrolyzed with various enzymes. CME, *Cordyceps militaris* mushroom extract; CMB, *Cordyceps militaris* mushroom extract hydrolyzed with bromelain; CMF, *Cordyceps militaris* mushroom extract hydrolyzed with flavorzyme; CMBF, *Cordyceps militaris* mushroom extract hydrolyzed with a mixture of bromelain:flavorzyme. ^a,b^ Mean values with different superscripts are significantly different among *Cordyceps militaris* mushroom extract treatments (p<0.05).

**Figure 3:**
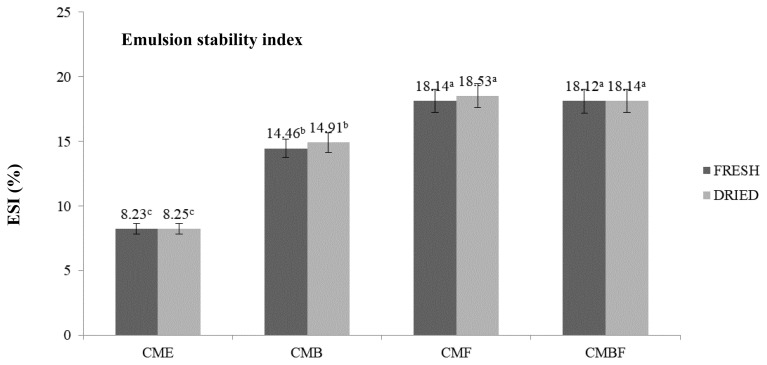
Emulsion stability index of the *Cordyceps militaris* mushroom extract hydrolyzed with various enzymes. CME, *Cordyceps militaris* mushroom extract; CMB, *Cordyceps militaris* mushroom extract hydrolyzed with bromelain; CMF, *Cordyceps militaris* mushroom extract hydrolyzed with flavorzyme; CMBF, *Cordyceps militaris* mushroom extract hydrolyzed with a mixture of bromelain:flavorzyme. ^a–c^ Mean values with different superscripts are significantly different among *Cordyceps militaris* mushroom extract treatments (p<0.05).

Figure 2 and 3 display the emulsifying capacity including the ESI and EAI. This assay evaluate the capability of protein to contibute as stabilizer in the newly created emulsion, and its capacity to emulsify oil. These two main parameters are a crucial importance to develop the protease and protein hydrolysate, since it is highly correlate with the power of protein to remain stable after mixing with certain solution without flocculation, coalescing, or creaming [19]. In addition, understanding the stability and activity index from the emulsion help to comprehend the product shelf life, stability prediction related to the appearances and textures, as well as formulation optimization and quality assurances [16]. This study observed a notable differences between the crude CM extract with that of hydrolyzed with various enzymes. Regardless of the utilized enzymes, the CM mushroom extract powder yielded in a significantly higher ESI and EAI (p<0.05).

### Shear force value and myofibrillar fragmentation index

In this study, dipping marination on spent hen breast was set at 4% according to the best result during preliminary experiment (data not shown). As shown in Table 5, a significant interaction between treatment with various CM mushroom hydrolysates and raw material used to generate mushroom hydrolysates was observed (p<0.05). Breast meat treated under fresh CM protease that was previously hydrolysate using bromelain (CMB) displayed the lowest kgf value at 2.21, followed by CMBF (2.28), CMF (2.42), and CME (2.74) respectively (p<0.05). Similar result for shear force was also observed under dried CM protease. The order from the most tender to the lowest was CMB (2.72 kgf), CMBF (2.71 kgf), CMF (2.80 kgf), and CME (2.82) (p<0.05), respectively. In addition, this study revealed that the CM protease subjected to hydrolyzation using fresh mushroom possess more significant effect for tenderization in comparison to that of dried ones (p<0.05).

### Content of adenosine 5′-monophosphate and inosine 5′-monophosphate

The total amount of both AMP and IMP within breast samples after subjection to various CM extract and hydrolysates are displayed in Table 6. The adenosine monophosphate contributes for the changes in proteolytic activities and other tenderization indexes in chicken meat by maintaining the up- and down-regulation of tenderness-related enzymes (calpain, cathepsin-B, and caspase-3) [8]. Therefore, it is essential to understand its dynamic presence within the meat samples. This present study recorded a significant improvement in AMP concentration after hydrolyzation treatment. Regardless of the hydrolyzing enzymes, the content of AMP in fresh hydrolysate groups displayed notably higher concentration compared to that of non-hydrolysate ones (CME) (p<0.05).

### Collagen content

After marination for 1 hour using various mushroom hydrolysates, the collagen content of spent hen breast was evaluated. As indicated by Table 6, regardless of the mushroom hydrolysates, the total and insoluble collagen experienced no alteration (p>0.05).

## DISCUSSION

The increase of protein content found by this study may be due to the used enzymes that were able to extract and cleave intact proteins into liberated peptides, which increased the amount of peptide in solution and improved protein percentage [25]. The considerable protein content of CM mushrooms which are edible, makes them an intriguing experimental subject for generating either protease or protein hydrolysate. Since, the protein content determines further functional utilization. Additionally, it was mentioned that the protein-digesting and flavor intensifying protease is largely dependent on the raw material’s high protein concentration [14]. Following hydrolyzation, different raw materials employed in the proximate composition did not significantly differ from one another (p>0.05). Regarding the proximate composition of the dried CM mushroom, this study clarified a prior reportthat suggested the nutritional value of the CM mushroom could degrade after drying at particular temperature, primarily as a result of the loss of carbohydrate and some water soluble vitamines. This occurrence was well reported in fruiting body or mycelial biomass of the CM mushroom [23].

The findings of this study on instrumental color were consistent with those of a recent study by Ang and Ismail-Fitry [16] which discovered that bromelain-enhanced enzymatic hydrolysis reduced the lightness value of shiitake, oyster, bunashimeji, and enoki mushrooms. Additionally, it supports a work done in squid head hydrolysate by Sukkhown et al [15]. The increased amount of liberated peptides from the enzymatic hydrolysis process was believed to be the cause of dark-brown color. This condition further affect the luminosity of the extract solution into the darker color [26]. The CM mushroom protease produced from the fresh raw materials that was hydrolyzed with bromelain recorded the highest score (74.01%) among the samples. Furthermore, the fresh CM mushroom group displayed a substantially greater DH percentage in comparison to that of the dried one (p<0.05). Other functional features, such as yield percentage and nutritional content are significantly influenced by the DH percentage [14]. As also observed by this study, the higher DH percentage tended to produce higher yield percentage of the CM mushroom protease. This study confirms earlier report by Sukkhown et al [15] in squid head hydrolysate, wherein increased solubility and intact protein cleavage are indicated by a significantly higher score for DH percentage. This circumstance encourages the more release of functional peptides and increases more available bonding opportunities between the amino and carboxyl groups of amino acids, consequently intensifies the concentration of short chain peptides in solution, thus restrain the markedly higher yield percentage even after enzymatic hydrolysis process [27].

There are possibly two primary reasons for the drop of CM mushrooms pH value, first, the increased generation of the acid-releasing amino acid, such as glutamic and aspartic acid from both endo- and exopeptidase activity during enzymolysis on CM mushroom intact protein. Second, due to the loss of free H+ ions produced when links between amino acids are cleaved. The findings of this study were in agreement with those of Ang and Ismail-Fitry [16], in which various mushrooms that hydrolyzed with bromelainand enzymes resulted in robust endopeptidase activity [14]. Following hydrolysis, there is a significant interaction between pH-declining phenomena and the total soluble solid [28]. Since, the destruction of non-covalent bonds during enzymolysis process impacts in protein denaturation, thus, the intensifies the amount of soluble protein exist within the solution and the lower pH value of solution will be observed [29].

This present study observed the papain to had the highest enzymatic activity. Nevertheless, a higher proteolytic enzyme activity does not necessarily contribute to the intended results for meat tenderization, as mushy texture and overtenderization of the meat are both possible outcomes. Papain as an example, which has been well-studied for having a wide range of proteolytic enzyme activity, are characterized to capable of hydrolyzing both connective tissue and myofibrillar proteins [30], which results in unfavorable quality attributes, such as overtenderized meat, bitter taste, and off-odor [31]. Therefore, these findings suggest that the application of the exogenous protease derived from CM mushroom has a milder tenderizing effect to ensure minimal side effects during postmortem tenderization [32].

By means of DPPH radical scavenging activity, the antioxidant activities of the CME that was prepared with various enzymes were carefully evaluated. The antioxidant measurement using DPPH assay is one of widely used spectrophotometric techniques based on the color alterations. The stable purple color of the DPPH solution would experience reduction and changed into yellow color after reaction with the potentially enriched-antioxidative substances [33]. With respect to the utilized enzyme, CM mushroom extracts prepared with bromelain showed the highest capability to diminish the DPPH radical. The potent endopeptidase activity from bromelain might cleave the inner chain of the intact protein, resulting in a more sulfur-containing amino acids (cystein, methionine, taurine, and glutathione) in comparison with the exopeptidase ones [34]. This finding on the antioxidant activity after hydrolysation of CM with distinct peptidases were in agreement with the previous study by Gao et al [14] in morel mushroom, in which endopeptidase hydrolysed extract presented higher antioxidant capacity. In addition, there was no differences observed in the emulsifying capacity profiles that includes the ESI and EAI, recorded under Table 3 and 4 between fresh and dried condition of CM mushroom used. The more formation of the shorter chain peptides resulted from the intact protein degradation by the enzymes was mentioned to induce an improved efficiency of the protease powder to reduce the interfacial tension and stabilize emulsion due to the rapid diffusion and adsorbility at the interface [35].

With regard to the fragmentation of myofibril after treatment, hydrolyzation using various enzymes tended to donate significant effect on MFI (p<0.05). As seen on Table 5, all hydrolysate treated groups displayed significantly higher fragmentation score compared to that of non-hydrolysate ones (CME). In addition, breast sample that was marinated with fresh mushroom extracts resulted in a notably higher MFI compared to that marinated with the dried one (p<0.05). During postmortem, a significant tenderization activity can be reflected by the fragmentation of myofibrils. The MFI measures the extent of architectural alteration of various protein components, including desmin, nebulin, troponin-t, vinculin, and titin [36,37]. The structural degradation of these key proteins is commonly caused by both activity of endogenous and exogenous enzymes [38] and nucleotide concentrations [36].

A significant improvement in spent hen meat tenderness following treatment with CM hydrosates indicated a broader binding site that the hydrolysate could use as substrate. It was reported that the CM mushrooms are equipped with metalloprotease that could bind to specific area in myofibrillar protein [39]. The hydrolyzation using bromelain and flavourzyme possibly allows the CM protease to degrade more variation of substrates.

This present study recorded no notable differences on chicken breast samples treated using dried mushroom hydrolysates. In addition, with regard to that of IMP within the breast sample, this study revealed a significant effect of hydrolyzation, wherein all hydrolysate treated groups displayed notably higher IMP content than that of CME ones (CME) (p<0.05). No significant differences were observed concerning enzyme variation used for hydrolyzation (p>0.05). CM mushrooms are enriched with sufficient content of AMP. It occupies the second largest portion after cordycepin. Using HPLC, we quantified the average content of AMP in utilized mushroom was at 0.096 mg/g. The introduction of exogenous AMP into meat environment were proven to enhance its existence within the muscle [8,21]. Following its permeation into muscle inner environment, however, AMP will soon experience an enzymatic reaction by adenosine deaminase and converted into adenosine and IMP. This clarifies the finding by this study, in which the IMP in the utilized chicken breast sample was detected at high concentration similar to that of AMP results’ trend. It was suggested that inosinic acid including AMP and IMP play a significant role for the improvement of flavour and texture in meat products [21,40]. It serves as a highly effective salt to provide both umami taste and enhance texture perception toward meat muscle. The result of this study suggested the hydrolyzation of CM mushroom has potential in providing spent hen meat with a richer flavour profile accompanied by decent texture attributes.

Related to the content of breast meat sample’s collagen, results of this study indicate a mild proteolytic activity owned by CM mushroom hydrolysates. During their effort to tenderize muscle protein, plant proteases possess distinctly different mechanisms. They are differentiated by means of protein substrate they can degrade and optimum condition during postmortem tenderization [41]. Although its limitation to penetrate toward muscle environment, protease enzyme from papaya (papain) are characterized as a robust enzyme due to its broad spectrum of substrate, including key proteins, myofibrillar proteins, as well as connective tissue [4]. Its strong activity, however, is commonly associated with over tenderization and pungent aroma. The result of this study indicates that the contribution of CM mushroom hydrolytes for tenderization of spent hen breast was limited to only myofibrillar protein and not toward collagen.

## CONCLUSION

In conclusion, this research demonstrated that enzymatic hydrolysis, particularly with bromelain, enhances the functional properties of *Cordyceps militaris* mushroom protease. The process not only increased protein content and altered color profiles but also significantly improved antioxidant activity when fresh mushrooms were used. Moreover, marination on breast meat samples with hydrolyzed CM protease positively affected the texture and flavor profiles of spent hen breast meat without altering collagen concentration. Therefore, bromelain hydrolysis of CM protease is recommended for its potential to improve taste and texture in meat products.

## Figures and Tables

**Table 1:** Proximate composition of the *Cordyceps militaris* mushroom extract hydrolyzed with various enzymes

Variables	Condition	Treatments^1)^					SEM	p-value		
	
		CM	CME	CMB	CMF	CMBF		Condition	Enzyme	Interaction
Moisture (%)	Fresh	77.43^Aa^	28.35^Ab^	28.76^Ab^	29.48^Ab^	29.71^Ab^	3.03	<0.05	<0.05	0.28
	Dried	45.01^Ba^	28.15^Ab^	28.81^Ab^	28.11^Ab^	28.89^Ab^	2.11			
	SEM	1.11	0.89	0.22	0.09	0.21				
Crude fat (%)	Fresh	1.01^a^	0.98^b^	1.16^a^	1.21^a^	1.21^a^	0.03	0.16	<0.05	0.91
	Dried	1.42^a^	0.96^b^	1.09^a^	1.11^a^	1.15^a^	0.01			
	SEM	0.14	0.02	0.15	0.18	0.07				
Crude protein (%)	Fresh	28.55^b^	30.67^a^	30.92^a^	30.32^a^	30.12^a^	0.52	0.18	<0.05	0.46
	Dried	27.81^b^	30.55^a^	30.97^a^	30.19^a^	30.11^a^	0.21			
	SEM	0.92	0.11	0.23	0.14	0.30				
Ash (%)	Fresh	5.30	5.05	5.09	4.99	5.22	0.02	1.07	0.98	1.25
	Dried	5.06	5.08	5.01	4.86	5.33	0.04			
	SEM	0.08	0.09	0.12	0.06	0.05				

SEM, Standard error of the means.

1) CM, *Cordyceps militaris* mushroom; CME, *Cordyceps militaris* mushroom extract; CMB, *Cordyceps militaris* mushroom extract hydrolyzed with bromelain; CMF, *Cordyceps militaris* mushroom extract hydrolyzed with flavorzyme; CMBF, *Cordyceps militaris* mushroom extract hydrolyzed with a mixture of bromelain:flavorzyme.

a,b Mean values within the same row with different superscripts are significantly different among *Cordyceps militaris* mushroom extract treatments (p<0.05).

A,B Mean values within the same column with different superscripts are significantly different between *Cordyceps militaris* mushroom condition (p<0.05).

**Table 2:** Instrumental color of the *Cordyceps militaris* mushroom extract hydrolyzed with various enzymes

Variables	Condition	Treatments^1)^					SEM	p-value		
	
		CM	CME	CMB	CMF	CMBF		Condition	Enzyme	Interaction
L*	Fresh	72.11^Aa^	64.98^Ab^	58.02^c^	56.17^d^	56.33^d^	1.11	<0.05	<0.05	0.12
	Dried	68.36^Ba^	63.21^Bb^	57.91^c^	56.12^d^	56.11^d^	0.95			
	SEM	2.02	1.86	1.05	1.05	1.16				
a*	Fresh	7.12^c^	10.22^b^	13.21^a^	14.03^a^	13.94^a^	1.12	0.09	<0.05	0.29
	Dried	6.31^c^	10.11^b^	13.17^a^	13.98^a^	13.89^a^	0.58			
	SEM	1.09	0.92	0.87	1.06	0.79				
b*	Fresh	30.14^Aa^	26.85^Ab^	21.56^Ac^	20.65^Ac^	20.13^Ac^	1.37	<0.05	<0.05	0.32
	Dried	26.85^Ba^	26.33^Aa^	21.23^Ab^	20.97^Ab^	20.02^Ab^	1.07			
	SEM	1.14	1.20	0.93	0.72	0.69				

SEM, standard error of the means.

1) CM, *Cordyceps militaris* mushroom; CME, *Cordyceps militaris* mushroom extract; CMB, *Cordyceps militaris* mushroom extract hydrolyzed with bromelain; CMF, *Cordyceps militaris* mushroom extract hydrolyzed with flavorzyme; CMBF, *Cordyceps militaris* mushroom extract hydrolyzed with a mixture of bromelain:flavorzyme.

a–d Mean values within the same row with different superscripts are significantly different among *Cordyceps militaris* mushroom extract treatments (p<0.05).

A,B Mean values within the same column with different superscripts are significantly different between *Cordyceps militaris* mushroom condition (p<0.05).

**Table 3:** Physicochemical properties of the *Cordyceps militaris* mushroom extract hydrolyzed with various enzymes

Variables	Condition	Treatments^1)^				SEM	p-value		
	
		CME	CMB	CMF	CMBF		Condition	Enzyme	Interaction
Yield (%)	Fresh	7.02^Ac^	9.04^Aa^	7.98^Abc^	8.12^Ab^	0.03	<0.05	<0.05	0.09
	Dried	5.12^Bb^	7.11^Ba^	7.09^Aa^	6.55^Ba^	0.01			
	SEM	1.89	1.77	1.03	1.52				
DH (%)	Fresh	18.67^Ac^	74.01^Aa^	70.75^Ab^	70.21^Ab^	5.21	<0.05	<0.05	0.08
	Dried	17.80^Ac^	50.68^Ba^	39.28^Bb^	40.12^Bb^	4.79			
	SEM	1.09	2.68	3.91	3.88				
pH	Fresh	6.47^a^	5.31^c^	5.42^b^	5.40^b^	0.21	0.18	<0.05	0.57
	Dried	6.47^a^	5.35^c^	5.39^b^	5.38^b^	0.17			
	SEM	0.85	0.76	0.88	0.52				
Total solule solid (°Brix)	Fresh	3.83^Ac^	8.73^Aa^	7.87^Ab^	7.85^Ab^	0.30	<0.05	<0.05	0.11
	Dried	4.13^Ab^	5.87^Ba^	4.83^Bab^	4.90^Bab^	0.21			
	SEM	0.97	1.04	1.01	0.94				

SEM, standard error of the means; DH, degree of hydrolysis.

1) CM, *Cordyceps militaris* mushroom; CME, *Cordyceps militaris* mushroom extract; CMB, *Cordyceps militaris* mushroom extract hydrolyzed with bromelain; CMF, *Cordyceps militaris* mushroom extract hydrolyzed with flavorzyme; CMBF, *Cordyceps militaris* mushroom extract hydrolyzed with a mixture of bromelain:flavorzyme.

a–c Mean values within the same row with different superscripts are significantly different among *Cordyceps militaris* mushroom extract treatments (p<0.05).

A,B Mean values within the same column with different superscripts are significantly different between *Cordyceps militaris* mushroom condition (p<0.05).

**Table 4:** Proteolytic enzyme activity of the *Cordyceps militaris* mushroom extract hydrolyzed with various enzymes

Variable	Condition	Treatments^1)^					SEM	p-value		
	
		Papain	CME	CMB	CMF	CMBF		Condition	Enzyme	Interaction
Enzyme activity (unit/mL)	Fresh	9.61^a^	2.90^Ae^	4.57^Ab^	3.89^Ad^	4.01^Ac^	0.10	<0.05	<0.05	0.24
	Dried		2.78^Ab^	2.38^Bb^	2.01^Bb^	2.54^Bb^	0.08			
	SEM		0.02	0.04	0.04	0.07				

SEM, standard error of the means.

1) CM, *Cordyceps militaris* mushroom; CME, *Cordyceps militaris* mushroom extract; CMB, *Cordyceps militaris* mushroom extract hydrolyzed with bromelain; CMF, *Cordyceps militaris* mushroom extract hydrolyzed with flavorzyme; CMBF, *Cordyceps militaris* mushroom extract hydrolyzed with a mixture of bromelain:flavorzyme.

a–e Mean values within the same row with different superscripts are significantly different among *Cordyceps militaris* mushroom extract treatments (p<0.05).

A,B Mean values within the same column with different superscripts are significantly different between *Cordyceps militaris* mushroom condition (p<0.05).

**Table 5:** Texture profile and myofibrillar fragmentation index of breast meat from spent chicken after treatment with *Cordyceps militaris* mushroom hydrolysates

Variables	Treatments^1)^				SEM	p-value		
	
	CME	CMB	CMF	CMBF		Treatment	Condition	Treatment × condition
Shear force value (kg)								
Fresh	2.74^Ba^	2.21^Bc^	2.42^Bb^	2.28^Bc^	0.00	<0.05	<0.05	<0.05
Dried	2.82^Aa^	2.72^Ab^	2.80^Aa^	2.71^Ab^	0.04			
SEM	0.02	0.03	0.03	0.02				
Myofibrillar fragmentation index								
Fresh	36.87^Ac^	48.14^Aa^	38.09^Ab^	47.24^Aa^	0.06	<0.05	<0.05	0.07
Dried	30.18^Ac^	42.10^Ba^	37.19^Ab^	40.59^Bab^	2.76			
SEM	1.17	1.04	0.98	1.12				

SEM, standard error of the means.

1) CME, *Cordyceps militaris* mushroom extract; CMB, *Cordyceps militaris* mushroom extract hydrolyzed with bromelain; CMF, *Cordyceps militaris* mushroom extract hydrolyzed with flavorzyme; CMBF, *Cordyceps militaris* mushroom extract hydrolyzed with a mixture of bromelain:flavorzyme.

a–c Mean values within the same row with different superscripts are significantly different among *Cordyceps militaris* mushroom extract treatments (p<0.05).

A,B Mean values within the same column with different superscripts are significantly different between *Cordyceps militaris* mushroom condition (p<0.05).

**Table 6:** Nucleotide and collagen contents of breast meat from spent chicken after treatment with *Cordyceps militaris* mushroom hydrolysates

Variables	Treatments^1)^				SEM	p-value		
	
	CME	CMB	CMF	CMBF		Treatments	Conditions	Treatments × conditions
AMP (mg/g)								
Fresh	0.41^Ab^	0.59^Aa^	0.61^Aa^	0.60^Aa^	0.03	<0.05	0.13	0.42
Dried	0.38^A^	0.43^B^	0.41^B^	0.44^B^	0.04			
IMP (mg/g)								
Fresh	1.05^b^	5.09^a^	4.89^a^	4.94^a^	0.11	<0.05	0.83	0.72
Dried	1.20^b^	5.17^a^	5.02^a^	4.87^a^	0.09			
Total collagen (mg/g)								
Fresh	2.02	2.19	2.11	2.24	0.17	0.46	0.78	0.69
Dried	2.11	2.07	2.15	2.20	0.16			
Insoluble collagen (mg/g)								
Fresh	1.72	1.64	1.81	1.69	0.13	0.91	0.43	0.67
Dried	1.89	1.77	1.80	1.76	0.08			

SEM, standard error of the means; AMP, adenosine 5’-monophosphate; IMP, inosine 5’-monophosphate.

1) CME, *Cordyceps militaris* mushroom extract; CMB, *Cordyceps militaris* mushroom extract hydrolyzed with bromelain; CMF, *Cordyceps militaris* mushroom extract hydrolyzed with flavorzyme; CMBF, *Cordyceps militaris* mushroom extract hydrolyzed with a mixture of bromelain:flavorzyme.

a,b Mean values within the same row with different superscripts are significantly different among *Cordyceps militaris* mushroom extract treatments (p<0.05).

A,B Mean values within the same column with different superscripts are significantly different between *Cordyceps militaris* mushroom condition (p<0.05).

## Data Availability

The datasets generated during the current study are available from the corresponding author on reasonable request.
